# A Century of Medical History in the County of Erie—1800–1900

**Published:** 1898-04

**Authors:** William Warren Potter

**Affiliations:** Buffalo, N. Y.


### BUFFALO MEDICAL JOURNAL.

VOL. XXXVII. APRIL, 1898. No. 9.

### Original Communications.

### A CENTURY OF MEDICAL HISTORY IN THE COUNTY OF ERIE—1800–1900.[1]

BY WILLIAM WARREN POTTER, M. D., Buffalo, N. Y.

*Pioneer Physicians—Medical Societies—Medical Colleges—Hospitals—Medical Journals—Medical Officers of the Civil War—Women Physicians—History of Homeopathy—Individual Members of the Profession.*

### INTRODUCTION.

THE importance of preserving accurate history of all kinds in an accessible form constantly increases as years advance. This observation has no more forceful application than to the medical men and medical institutions of the county of Erie. As our county grows older and busier, with its rapidly increasing population and business development, the early landmarks are getting swept away and soon it will be difficult or impossible to trace medical events with accuracy unless they are recorded from time to time with precision and detail.

Several attempts heretofore have been made to preserve such a history, fragments of which are to be found scattered here and there through volumes, magazines and library papers. In arranging this record these have all been carefully searched, many of which have aided materially in its preparation. The purpose of this work is to bring the record forward to meet the conditions of the present period, giving data that have been verified and material that would seem of sufficient importance to justify its publication.

1. From "Our County and its People." Republished as a serial in this magazine by permission of the Boston History Company.

Moreover, as the century is drawing near its close the time would seem opportune to present in compact form, convenient for reference, a record of the principal events with which the medical profession in Erie county has been identified during the last hundred years. A grouping of these dates and divisions according to county boundary surveys appears necessary in order to reach the genesis of Erie county through hereditary lines.

While, therefore, the medical history of Erie county proper really begins when its present territorial lines were erected—namely, April 2, 1821, yet there is a valuable chapter that must be recorded relating to medical men of the period during which it was included within the boundaries of Niagara county.

### THE PIONEER PHYSICIANS.

*Cyrenius Chapin.*—Though there is very little medical history in this region prior to 1800 that can be grouped or made available, yet with the birth of the XIX. century, or while it was yet in its swaddling clothes, there came hither a man who has left the stamp of his forceful character impressed upon the events of that period so strongly that neither time nor tide nor any other thing can efface it. It is with the advent of this man that the medical history proper of this region may be said to have commenced.

Cyrenius Chapin was born at Barnardstown, Mass., February 7, 1769. There is very little known of his early life, but he studied for his profession with his eldest brother, Dr. Caleb Chapin, at his birthplace. He completed his studies in 1793 and soon afterward married Sylvia Burnham, also of Barnardstown. He practised medicine several years at Windhall, Vt., then removed to Sangerfield, Oneida county, N. Y., and came to Buffalo in 1801, a place which had then begun to attract immigration. It was called New Amsterdam when laid out in 1801 by the Holland Land Company, but the name was changed to Buffalo in 1808.

The Holland Land Company had lately established its agency at Buffalo, but the village had not yet been surveyed and so a project that Dr. Chapin had partially negotiated with Joseph Ellicott, the company's agent, for the purchase of a township of land including the site of Buffalo fell through and he returned to Sangerfield. Finally, however, in 1803, Dr. Chapin returned to Buffalo, bringing his family with him, but he was obliged to locate temporarily at Fort Erie in Canada, as he was unable to obtain a suitable home in Buffalo.

Dr. Chapin soon acquired a large practice on both sides of Niagara river. On the Canadian side there was wealth, but on this the people were poor; hence his income was principally derived from Canada. He rode hundreds of miles in all directions seeking his patients on horseback, guided through the forest by blazed trees and other methods of convenience and safety then in vogue. He established the first drug store in Buffalo, often furnishing medicine as well as food gratuitously to his needy patients. In 1806, he secured the title to a tract of land at the corner of Main and Swan streets, running through to Pearl street, for the sum of $150, on which he established his home and removed his family hither from Fort Erie. Chapin Block, which appropriately bears the name of its original purchaser, now stands on a portion of this lot.

Between 1806 and 1812 Dr. Chapin was busily engaged in the practice of his profession and in helping to improve the now growing village of Buffalo. He was the foremost man of affairs in this region and commanded the respect even of the Indians, who looked up to him as the great medicine man. It is related that when he lost an only son the Indian chiefs sent delegates to express their sympathy and who formally attended the funeral.

When, in 1812, war was declared with Great Britain, Dr. Chapin made haste to offer his services to the United States government. He raised a company of volunteers and served in the capacity of captain, major, lieutenant-colonel and surgeon. This is not the place to record Dr. Chapin's military career, but one or two incidents of importance may be noted with propriety. On June 24, 1813, having now been commissioned lieutenant-colonel, he was sent on a reconnoissance by Colonel Boestler, U. S. Army. At a point a few miles west of Queenstown his entire command was captured and marched to Fort George, where they were retained under guard. On July 12th, having been ordered to Kingston, they embarked in two open boats, strongly guarded. When within a few miles of their destination the boats approached each other, Colonel Chapin gave a preconcerted signal, his men rose and overpowered the guards, the boats were turned about, and after a night of toil at the oars his British captives were delivered to the commandant at Fort Niagara as prisoners of war.

Another incident will serve to indicate the character of the man. On December 30, 1813, the village of Buffalo was burned by the British and Indians. Colonel Chapin defended a position he had taken up at Black Rock until but five men of his command remained. He then retreated to Buffalo, where everything was in confusion. Mustering a few men and boys, he set about protecting the women and children, who had been left to care for themselves while their husbands and fathers were on duty at Black Rock. Colonel Chapin found a six-pounder cannon that he mounted upon wagon wheels and with it and his small force made a stand on Niagara street. He thus sought to delay the advance of the enemy until the women and children could escape, but after a few discharges of the gun its extemporised carriage broke down and the six-pounder became disabled.

Observing that further resistance was useless, Colonel Chapin tied a white handkerchief to his sword and rode out to make terms with the exultant, advancing foe. He agreed on his part to surrender all public property, arms and munitions of war. The enemy agreed to allow the women and children to remain unmolested and to protect as well as to respect private property. The British officer with whom the treaty was concluded violated the agreement almost immediately, permitting the Indians to burn the village. In the conflagration but two houses escaped destruction, which served as monuments to the perfidy of the British officer. Although Colonel Chapin failed to save the village, he gained for the inhabitants valuable time in which to escape and, unmindful of self, he surrendered himself a prisoner to protect his people from the vengeance of the British and Indians. He was taken to Montreal, where he was held a prisoner for nine months. Meanwhile, after the desolation of his home by fire, his family went to Canandaigua, where he visited them on his release. He then returned to Buffalo, where he was appointed surgeon to the military hospital. When relieved of this duty he located at Geneva, N. Y., but in 1818 he returned to Buffalo to reside permanently. He engaged in active professional practice, took a prominent place in medical societies and in public affairs and never lost interest in the welfare of the community. He died February 30, 1838, aged sixty-nine years, and was buried in the village cemetery with military honors.

*Daniel Chapin.*—Daniel Chapin came to this vicinity, from East Bloomfield, N. Y., in 1807. He established himself on a farm a few miles distant from the village of Buffalo, which property was afterward owned by Elam R. Jewett, on North Main street. Dr. Daniel Chapin resided in a log house, which was located in the rear of the present homestead. This latter was built by his son, Colonel William W. Chapin, and is now occupied by Mrs. Elam R. Jewett. Daniel Chapin, like his namesake, Cyrenius Chapin, was a man of strong character and left the stamp of its forcefulness on his environment. He was a graduate of Yale college, a man of cultivation and a physician of great skilfulness, who commanded the respect of his neighbors and colleagues. Between Drs. Daniel and Cyrenius Chapin, however, there prevailed a wordy but harmless rivalry, amusing to others, but not of serious consequence. Both were strong men : strong in their preferences, strong in their hatreds, but both contributed materially to the establishment of medical practice on a sounder and more scientific basis, details of which will be recorded later.

Daniel Chapin was accustomed to visit his patients on foot, with dog and gun, even traveling as far as Niagara Falls in this way, going one day and returning the next. He died in 1821, aged about sixty years. His death was at least partly induced by exposure in the practice of his profession, the hardships of those days being often extreme and perilous.

*Ebenezer Johnson.*—In 1809 another physician of prominence appeared in Buffalo, who has left a name deeply interwoven with its early history. Ebenezer Johnson came here from Cherry Valley, N. Y., established himself as a practising physician and soon took a prominent place in the affairs of the little frontier village. To these three men—the two Drs. Chapin and Dr. Johnson—Buffalo will ever remain indebted for the parts they performed in those early days. These pioneer physicians were more than ordinary men. They were not only able physicians who were beloved by their patients, but they were also men who contributed largely toward laying the foundation of successful business enterprises that now make the city so famous.

Dr. Johnson established a drug store in connection with his medical practice, as Cyrenius Chapin had done before him—a fashion not uncommon in those days. Dr. Johnson was also, for a time, associated in business with Judge Wilkeson and afterward he established a bank. He accumulated a fine property and was elected the first mayor, when in 1832 Buffalo became a city, and three years later was again chosen to the same office. In 1815, and again in 1828, he was elected surrogate of Erie county. He lived in a handsome house on Delaware avenue, now a part of the Female Academy. In the financial revolution of 1837 he met reverses, losing a large part of his property. He went to Tennessee with his brother to look after their interests in an iron mine. He died at Tellico Plains, Tenn., February 8, 1849, aged sixty-three years.

*Josiah Trowbridge.*—In the spring of 1811 the medical contingent of this new region received a strong reinforcement in the person of Dr. Josiah Trowbridge, who came on horseback from Vermont, with a lawyer friend by the name of Walker. Following the example of Dr. Cyrenius Chapin, and for a similar reason—namely, lack of suitable living accommodations at Buffalo, Dr. Trowbridge took up his abode at Fort Erie.

The declaration of war the following year caused him to return to the United States, but his heart was in the queen's dominions, as he had formed an attachment for one of Her Majesty's subjects. On the 19th of September, 1814, he crossed the Niagara river, captured Margaret Wintermute, and was married in Buffalo on the 22d of the same month. Dr. Trowbridge gave the government hearty support during the war of 1812, though he was not a sympathiser with the war party. He became a member of a volunteer artillery company, in the ranks of which he served. He was fond of his gun, and one day when shooting ducks on Strawberry Island in company with an officer, Lieutenant Dudley, of Perry's fleet, together with some other friends, he and his companions were surprised and captured by the British. He was taken to Fort George, where the Indians threatened him with bodily harm, but the officers and chiefs interfered, thus preventing a prospective massacre. After a few days' detention Dr. Trowbridge and his companions were released, whereupon they returned to Buffalo after a tedious journey on foot. Dr. Trowbridge continued in active practice until 1836, having accumulated a handsome property. This he lost in 1837 when financial reverses came to all. He was elected mayor of Buffalo this same year. He did a large consultation practice until 1856, when failing health compelled him to relinquish it. He died September 18, 1862, aged seventy-seven years.

*John E. Marshall.*—The next medical man of prominence to locate at Buffalo was Dr. John E. Marshall. He came in 1815; but he had practised previously at Mayville, N. Y., and was the first clerk of the county of Chautauqua (1811), having been appointed by Gov. Daniel D. Tompkins. Dr. Marshall was born at Norwich, Conn., March 18, 1785, and was licensed to practise medicine August 3, 1808, by the Connecticut state medical society.

He practised for six years at Mayville, that is, from October, 1809, to March, 1815, and married, September, 1810, Ruth, daughter of Orasmus Holmes, one of the early pioneers of Chautauqua county. Dr. Marshall was appointed surgeon of the Second Regiment New York State Militia by Col. McMahon, April 15, 1812, and joined the regiment at Buffalo about December 30, 1813. He served for five months on the frontier, when his regiment was disbanded and he returned home. On August 1, 1814, he was again ordered to report for duty. There were many sick during August and September of that year and in their care Dr. Marshall, who was senior medical officer on hospital duty, himself fell sick and was obliged to return to his home. It is believed that for many years he continued to suffer from this camp sickness. He resumed his duties late in the fall, soon after which his regiment was discharged. After his removal to Buffalo, in March, 1815, he began to acquire fame as a medical practitioner and he was equally respected as a citizen.

He was appointed clerk of Niagara county (then embracing the present county of Erie) in 1818, and in 1832 he was health physician of Buffalo. He was treasurer of the county medical society in 1826, 1827 and 1828 and was president in 1830. He died December 27, 1838, of pneumonia after a brief illness.

These five stalwart physicians—the two Drs. Chapin, Johnson, Trowbridge and Marshall,—constituted a phalanx that served as a basis for the future medical history of Buffalo and Erie county. The history of this epoch is not one of medical organisations, institutions, societies, hospitals or colleges, but rather a history of the individuals who composed the medical profession of the period and the locality. It is for this reason that the history of medicine here up to 1821 is necessarily a grouping of biographical sketches.

### The Medical Society of the County of Erie.

In November, 1805, a movement was begun in Saratoga county looking toward the organisation of the medical profession into a fixed and definite body, for the purpose of improving its status and to resuscitate it from the obscurity and ill-repute that ignorance was fast consigning it. A meeting was held during which committees were appointed and a resolution passed inviting the coöperation of the neighboring counties of Montgomery and Washington. An adjournment was had until January, 1806, at Balston, at which time a memorial to the legislature was adopted and a committee consisting of Drs. Fitch, of Washington, Stearns, of Saratoga, and Sheldon, of Montgomery, was appointed to present it. Two of the committee, Dr. Stearns and Dr. Sheldon, attended the next session of the legislature as members, when, fortunately, Dr. Sheldon was chosen speaker.

Though the committee referred to was charged with representing only the three counties named, it assumed the responsibility of extending the privileges of the proposed medical practice act over the entire state so that all the counties might be included in its provisions. The memorial was presented to the assembly in February, 1806, and referred to a committee consisting of the following-named members : William Livingston and Isaac Sargeant, of Washington, Gordon Huntley, of Otsego, John Ely, of Greene, and Joel Frost, of Westchester. It so happened that the majority of the committee were physicians and after considering the bill, which now contemplated a general law applicable to the entire state, reported it favorably to the house. Here it was destined to encounter bitter opposition, for then, as now, there were plenty of men to array themselves against the advance of medical education. But, supported by the speaker, the committee and other members of powerful influence, the bill finally passed, though even at last it might have failed had not, at a critical juncture, William P. Van Ness, a forceful speaker and a skilful parliamentarian, lent the aid of his powerful influence in a speech noted for its eloquence and argumentative weight. The bill became a law on April 4, 1806, and on the first Tuesday of July, three months afterward, about twenty county societies were organised. Within the next two years nearly every county in the state had its medical society.

Under the provisions of this law the medical society of the state of New York was organised on the first Tuesday of February, 1807, which consisted of one delegate from each county society. Among the provisions of the statute was a section giving the societies control of the licensing of physicians after due examination, which was among the first efforts in the country to give to the medical profession an honorable and legal standing in community.

The control of examining and licensing was subsequently lost to the state, and we shall see presently how important a part the medical society of the county of Erie played in bringing about its restoration.

Although the state medical society was organised in 1807, as we have seen, it was not until 1817 that Niagara county, of which Erie then formed a part, was represented in it by accredited delegate. Dr. James H. Richardson was the first delegate from Niagara county. He attended, presented his certificate of delegation and took his seat in 1817. It does not appear that Dr. Richardson attended more than one session of the state society.

Erie county, as we have previously remarked, was a part of Niagara county from 1808 to 1821 when the division was made. In Niagara county attempts were made to organise a medical society as early as 1808 or 1809, but owing to differences of opinion among physicians, the unsettled state of society in general, the approaching difficulties with Great Britain and finally the war of 1812, no definite organisation was effected until 1816. The first delegate from the medical society of Niagara county to the medical society of the state of New York was Dr. James H. Richardson, as we have seen, who was sent on that duty in 1817.

After the county of Erie was set off in 1821 its medical society became entitled to a seat on its own account and Dr. Lucius H. Allen was appointed delegate. He appeared and took his seat in February, 1823. There is no record to show that he ever attended another meeting. Indeed, Erie county was not represented again in the state society until 1833, when Dr. Bryant Burwell was seated as delegate.

The medical society of the county of Erie was organised January 9, 1821, at the house of P. M. Pomeroy, in the village of Buffalo. There were twenty-four charter members, whose names were as follows : Daniel Allen, of Hamburg, Lucius H. Allen, of Buffalo, Cyrenius Chapin, of Buffalo, Thomas B. Clark, of Buffalo, Sylvester Clark, of Buffalo, Benjamin C. Congdon, of Buffalo, Jonathan Hoyt, of Aurora, Jonathan Hurlburt, of Buffalo, Daniel Ingalls, of Springville, Ebenezer Johnson, of Buffalo, William Lucas, of Buffalo, Charles McLowth, of Buffalo, John E. Marshall, of Buffalo, William H. Pratt, of Eden, Charles Pringle, of Hamburg, Elisha Smith, of Buffalo, Rufus Smith, of Aurora, Sylvanus S. Stuart, of Buffalo, Ira G. Watson, of South Wales, John Watson, of Aurora, James Woodward and Josiah Trowbridge, of Buffalo.

The following-named were elected officers : President, Cyrenius Chapin ; vice-president, Daniel Chapin ; secretary, John E. Marshall ; treasurer, Lucius H. Allen ; censors, Charles Pringle,

Sylvanus S. Stuart, Benjamin C. Congdon, Lucius H. Allen and John E. Marshall.

Many of these physicians had lived in Western New York for years before Erie county was set apart and had been members, as well as in some instances officers, of the medical society of the county of Niagara.

At the first meeting of the medical society of the county of Erie the president, Dr. Cyrenius Chapin, delivered an address in which, among other things, he inveighed against quacks, who, he said, did no end of harm, and he also deplored the inconsistency and cold ingratitude of the public toward the medical profession; and further, he affirmed, "the truth is too obvious to require illustration that our profession is far from maintaining the rank among the learned professions which its consequence demands." In the course of his address he called attention to the fact that the services of physicians were undervalued by the public, and he suggested that as they were not charitable institutions it was time to resolutely determine upon a total reformation.

In a public notice which he issued about this period Dr. Chapin stated that he felt it his duty to inform those indebted to him for professional services that the time had arrived when imperious necessity compelled him to make an immediate colleetion of his accounts. We quote from this notice as follows:

> It has too long been a prevalent idea with the public that the physician's bill is never to be paid and to call upon a patient, when restored to health and to the enjoyment of life by the skill and attention of his physician, for a reward for the services rendered is considered almost an insult and a hardship. . . . To relieve my own necessity I am compelled to resort to an immediate collection and this I shall do without discrimination. Those, therefore, who think it a duty to save the cost of prosecution will find it expedient to bestow immediate attention to this subject.

The question of finance was an important one to the society. Dr. Lucius H. Allen was its first treasurer, but he does not appear to have left a record of his service. The first treasurer's record obtainable is dated January 9, 1827, at which time Dr. John E. Marshall was treasurer. This report shows that the receipts for the previous year were $11 and the disbursements $8. In 1830 the treasury contained six shillings, while the debts amounted to $10.50. In struggling to maintain a cohesion of membership the society sought to enforce the attendance of its members. No person was legally entitled to practise medicine or surgery in Erie county, except he was a member of the society. It, therefore, became compulsory on him to join and if otherwise qualified the society had no right to refuse him admission. Likewise, there was no legal way to get out of the society, hence it was empowered by law to exercise all sorts of discipline. Among other rules, it imposed a penalty of $1 for absence at any meeting and some amusing incidents arose out of the endeavor of the treasurers to collect this fine.

At one of the sessions the following was adopted : " *Resolved*, That the treasurer be directed to collect outstanding dues from members—peaceably if he can, forcibly if he must." In response to his demands a large number of letters were received which indicated the unpopularity of the proceedings. One of these is deemed of sufficient interest for a synopsis of it to find a place in these pages. It was written by Dr. Bela H. Colegrove, of Sardinia, to Dr. Marshall, under date of June 11, 1838. In it Dr. Colegrove protests against being fined for nonattendance, because the rule is unjust and discriminates against members. He lived thirty miles from the place where the meetings are required by law to be held and to go there twice a year meant a sacrifice to him of some $15 or $20, or to pay a penalty which the city members could avoid by the sacrifice of so many pence. He did not think he ought to compromise the interest of the community where he resided from neglect caused by attending the meetings. He did not complain of the amount, but of the principle, and he would as soon and with equal justice make the penalty for nonattendance imprisonment in the county jail for a term of six months as to have it as it now is.

[*To be continued.*]

